# Decision Variables for the Use of Radioactive Iodine in Patients with Thyroid Cancer at Intermediate Risk of Recurrence

**DOI:** 10.3390/cancers16173096

**Published:** 2024-09-06

**Authors:** Samantha K. Newman, Armando Patrizio, Laura Boucai

**Affiliations:** Department of Medicine, Endocrinology Service, Memorial Sloan Kettering Cancer Center, New York, NY 10021, USA; kasss@mskcc.org (S.K.N.); patriza1@mskcc.org (A.P.)

**Keywords:** thyroid cancer, radioactive iodine, intermediate risk

## Abstract

**Simple Summary:**

The use of radioactive iodine (RAI) for the treatment of patients with thyroid cancer at intermediate risk of recurrence is controversial. Evidence to date has not conclusively proven that there are benefits of this strategy to survival or recurrence after surgery for thyroid cancer. We describe key elements that can help clinicians decide when to prescribe RAI to this group of patients. These include a thorough discussion of the purpose of RAI therapy, better prediction of recurrence risk, the use of tumor markers after surgery, the use of the genetic profile of the tumor when available, and a detailed evaluation of the adverse effects of RAI, patient medical problems, and patient preferences. Integration of these variables will ensure a more comprehensive assessment of the risks and benefits of RAI treatment with the ultimate goal of avoiding overtreatment and reducing harm.

**Abstract:**

The use of radioactive iodine (RAI) after total thyroidectomy for patients at the American Thyroid Association (ATA) who are at intermediate risk of recurrence is controversial. This is due to the lack of prospective randomized trials proving a benefit to recurrence or survival of RAI therapy in this group. In the absence of such evidence, clinicians struggle to recommend for or against this therapeutic approach which frequently results in overtreatment. This review describes key elements in the decision-making process that help clinicians more comprehensively evaluate the need for RAI therapy in patients with thyroid cancer at intermediate risk of recurrence. A clear definition of the purpose of RAI therapy should be conveyed to patients. In this sense, adjuvant RAI therapy intends to decrease recurrence, and ablation therapy is used to facilitate surveillance. Better stratification of the intermediate risk category into a low–intermediate subgroup and an intermediate–high-risk subgroup results in less heterogeneity and a more precise prediction of recurrence risk. The evaluation of post-operative thyroglobulin levels may prevent the overtreatment of low–intermediate-risk patients when their thyroglobulin level is <2.5 ng/mL. the integration of tumor genomics (when available) alongside pathologic features can enhance the ability of the clinician to predict iodine concentration in thyroid cancer cells. Finally, a detailed consideration of the adverse effects of RAI, patients’ comorbidities, and patient preferences will result in a patient-centered personalized approach. Systematic examination of these variables will ultimately provide a framework for making more educated decisions on the use of RAI in patients at intermediate risk of recurrence that will prevent overtreatment and minimize harm.

## 1. Introduction

The use of radioactive iodine (RAI) for the treatment of thyroid cancer has become the standard of care after a total thyroidectomy based on reports from the 1940s and 1950s documenting its efficacy [1,2,3], and based on decreased 30-year recurrence and cancer-specific mortality rates in patients with advanced thyroid cancer who have received RAI compared to those who have not [4,5]. Despite the lack of prospective randomized trials supporting the efficacy of RAI therapy in patients at intermediate risk of recurrence, its empiric use has widely disseminated based on prospective observational and registry studies documenting some benefit in high-risk patients [6,7,8,9].

The American Thyroid Association (ATA)’s intermediate risk category, which includes about 40–60% of the patients with newly diagnosed thyroid cancer [10,11] and is associated with a 3–30% chance of disease recurrence [12], represents a very heterogeneous group of individuals with very diverse tumor characteristics that may impact the clinician’s decision to recommend RAI therapy. Attempts have been made to recommend for or against the use of radioactive iodine in this category of patients with some non-randomized studies suggesting a benefit of RAI in decreasing recurrence particularly in elderly patients with aggressive histologies and a large-volume lymph node disease, whereas other studies showing no benefit of RAI in this group [13,14,15,16]. This controversy resulted in the ATA recommendation of selective use of radioactive iodine therapy in this group of patients. The European Thyroid Association (ETA) [17] agreed that RAI may be indicated for use in intermediate-risk patients with advanced age; aggressive histologies; increasing volume of nodal disease; extranodal extension of the tumor; multiple N1; and/or lymph node metastases outside the central neck. The Latin American Thyroid Society (LATS) categorizes patients with thyroid cancer into very low risk, low risk, and high risk of recurrence. It suggests that the use of RAI for ablative purposes could be considered in patients at low risk of recurrence, whereas adjuvant RAI therapy should be administered to all patients with high-risk thyroid cancer [18]. To date, clear guidance and recommendations are still lacking.

Since the publication of these guidelines [12,17,18], endocrinologists have improved their ability to stratify the group of patients with thyroid cancer at intermediate risk of recurrence. Better pre-operative imaging has resulted in improved surgical outcomes, and more precise pathological evaluations better equip clinicians to predict recurrence risk. The availability of more sensitive tumor biomarkers, molecular testing, a better understanding of RAI side effects, and a detailed assessment of patients’ comorbidities and preferences can now more comprehensively inform the decision to proceed with radioactive iodine therapy. In this review, we describe the elements that are necessary to systematically consider before indicating RAI therapy for patients with thyroid cancer at intermediate risk of recurrence with the goal of minimizing risk without impacting survival.

## 2. Better Ability to Stratify the Intermediate Risk Group

The 2015 American Thyroid Association thyroid cancer guidelines [12] have improved our ability to classify patients at low and at high risk of recurrence compared to prior versions of this three-tier approach [19]. However, up to two thirds of patients with newly diagnosed differentiated thyroid carcinoma still fall in the intermediate-risk strata [10,11] which constitutes a very diverse group of patients with a wide range of cancer recurrence risk. In this sense, patients with extensive vascular invasion (>4 foci) are at the highest risk of recurrence and distant metastatic potential (15–30%) [20]. Similarly, patients with extensive cervical lymphadenopathy approach a 20% risk of recurrence, while patients with minimal extrathyroidal extension only experience recurrence in 3–8% [21] of cases, and yet all three groups of patients are still considered within the intermediate-risk category. The recognition of this differential recurrence risk has resulted in the 8th Edition of the AJCC/TNM guidelines no longer considering the pathologic finding of minimal extrathyroidal extension as a clinically relevant determinant of staging [22]. Moreover, additional tumor and patient characteristics including molecular events, patient’s age, body mass index, and others [23,24] may be essential in the future to refine the criteria that help predict risk of recurrence. The understanding of this disparate recurrence risk has allowed for a better sub-classification of the intermediate-risk group for the purpose of personalizing treatment decisions and avoiding overtreatment. It is now clear that within the intermediate-risk category of patients there is a subgroup of low–intermediate-risk patients with tumors that exhibit minimal extrathyroidal extension, or only a few or small size cervical lymph nodes metastatic for disease where the risk of recurrence can be estimated at 3–10%. For treatment purposes, no studies to date have conclusively shown that adjuvant radioactive iodine therapy can improve this already excellent recurrence risk [13] and ablative doses of this nuclear isotope may be used to facilitate follow-up of thyroglobulin levels as a thyroid cancer marker.

In the case of intermediate–high-risk patients with a larger size and number of lymph compromised nodes and/or extensive vascular invasion where the risk of recurrence approaches 20–30%, there are still no prospective randomized studies documenting the benefits of adjuvant RAI on recurrence rates, but in retrospective meta-analyses, a benefit of RAI on survival might emerge in this higher risk population [25]. Furthermore, if a decision is made to proceed with adjuvant RAI therapy for intermediate–high-risk patients, whether this treatment is administered immediately following surgery or delayed for a few months does not affect the response rate (pooled RR = 1.05, 95% CI: 0.96–1.15) or long-term overall survival of these patients (RR = 1.03, 95% CI: 0.81–1.31) [26].

Dynamic risk stratification [12] upon surveillance will help clinicians decide if RAI therapy is needed at a later stage. When thyroglobulin levels increase in the absence of structurally identifiable disease, patients are classified as having a biochemical incomplete response to therapy and they may elect to receive an ablative or an adjuvant dose of radioactive iodine to facilitate follow-up of their tumor markers. Conversely, patients in the intermediate–high risk strata who may have elected to avoid RAI therapy initially and exhibit an excellent response to therapy 3–5 years after their initial surgery can be reassured that RAI therapy may not have had a benefit on recurrence or survival in their case.

## 3. Role of Thyroid Lobectomy for Intermediate-Risk Patients

The recognition that there is a subgroup of patients with a lower risk of recurrence within the intermediate risk category, and that the benefits of adjuvant radioactive iodine therapy on recurrence are questionable in this subgroup has resulted in the adoption of a thyroid lobectomy as an alternative surgical intervention to total thyroidectomy for selected patients. In 2019, Liu et al. found that there was no significant difference in the 10-year recurrence-free survival (RFS) rate (77.4% vs. 80.2%, *p* = 0.622) or disease-specific survival (DSS) rate (97.2% vs. 98.4%, *p* = 0.554) among 341 matched pairs of patients treated with a thyroid lobectomy (TL) or total thyroidectomy (TT), respectively [27]. Similar results were recently reported by Xu among intermediate risk patients with clinical ipsilateral lymph node metastases (N1b disease) (5-year RFS rate between patients treated with TL and TT, 92.3% vs. 93.7%; *p* = 0.77) and the benefits of the lobectomy persisted when compared to propensity matched patients undergoing total thyroidectomy and radioactive iodine therapy (adjusted hazard ratio, 0.59; 95% CI, 0.14–2.41; *p* =  0.46) [28]. Conversely, Colombo et al. found that among intermediate-risk patients with larger tumors, a total thyroidectomy resulted in a higher proportion of excellent responses to therapy compared to patients undergoing a thyroid lobectomy (20/27, 74% vs 4/12, 33%, *p* = 0.016), but these two retrospective subgroups were not compared with propensity score matching [29]. Collectively, these data suggest that for a select group of predictably low–intermediate-risk patients, a thyroid lobectomy without a complete thyroidectomy or the addition of RAI may minimize surgical and medical complications without altering recurrence risk or survival outcomes.

## 4. Role of Post-Surgical Thyroglobulin in Intermediate-Risk Patients

Thyroglobulin levels are routinely checked following a total thyroidectomy with the intention of assessing the “completeness” of surgical treatment. When low, post-operative highly sensitive thyroglobulin measurements can be helpful in deciding whether to administer RAI or not in patients at intermediate risk of recurrence [30]. Chou and colleagues described that, after total or near total thyroidectomy, a non-stimulated thyroglobulin level <2.5 ng/mL might identify patients at low risk for persistent or metastatic disease [31]. The purpose of RAI therapy in this scenario must be conveyed and discussed with the patient. If the pathologic features of the tumor suggest an intermediate to high recurrence potential, we may administer RAI as adjuvant therapy, even in the presence of a low-post operative thyroglobulin level. But a low–intermediate-risk tumor with a low post-surgical thyroglobulin level may not necessitate RAI for either ablative or adjuvant purposes.

On the contrary, if the thyroglobulin level is significantly elevated after a total thyroidectomy, there is an increased likelihood of persistent thyroid cancer. To date, the quality of the evidence is too low to identify a unifying threshold thyroglobulin level above which persistent or metastatic disease can be suspected [31]. In this instance we should evaluate the surgeon’s experience [32], and re-stratify the tumor as low–intermediate or intermediate–high risk. In a low–intermediate-risk tumor with high thyroglobulin levels, RAI could be used for ablative purposes. In an intermediate–high-risk patient, the addition of RAI would not only be used to destroy remnant normal thyroid tissue, but also to target persistent thyroid cancer and to facilitate staging to identify the source of thyroglobulin production (Table 1). Whether adjuvant RAI therapy reduces the risk of recurrence in this scenario is still a matter of debate.

In the presence of post-operative thyroglobulin antibodies (Tg Abs) which occurs in patients with chronic lymphocytic thyroiditis, this biomarker is less effective when measured in a single time point, but it may be useful when tracked over time [12]. Prior studies [33] and a recent meta-analysis showed that the presence of Tg Abs or increasing trend after thyroid surgery confers a higher risk of cervical lymph node metastases (OR = 1.18 [CI 1.47–2.25]) and persistent/recurrent disease (OR = 2.78 [CI 1.55–4.98]) compared to patients without Tg Abs [34]. Furthermore, a decrease in anti-thyroglobulin antibodies over 47% or negative thyroglobulin antibodies in the first year post-surgery and RAI ablation predict a longer progression-free survival whereas a smaller decrease (<47%) or persistently elevated or increasing thyroglobulin antibodies are associated with persistent or recurrent disease [35]. Clinicians may choose to administer an ablative dose of RAI to facilitate follow-up of this thyroid cancer marker over time.

## 5. Role of Molecular Testing

The discovery of genetic alterations that constitutively activate intracellular MAPK signaling and in turn downregulate the expression of iodine metabolism genes has enhanced our understanding of the molecular underpinnings involved in the uptake and retention of radioactive iodine. Iodine concentration in thyroid cancer cells depends on iodine uptake, when the expression of the sodium iodine symporter is preserved on the cell membrane [36], and on iodine retention, due to preserved expression of thyroid peroxidase (TPO) involved in organification [37]. When MAP kinase pathway effectors like *BRAF* are mutated, the signaling through this pathway is augmented. This downregulates iodine metabolism genes, including the sodium iodine symporter and thyroid peroxidase, rendering cells less likely to take up and retain RAI [38]. This is particularly evident in patients with *BRAFV600E* mutations who are insensitive to downstream negative feedback regulation of this pathway and robustly suppress the expression of genes involved in iodine metabolism [39]. However, not all *BRAFV600E*-mutated papillary thyroid carcinomas behave in the same way. A subpopulation of patients with *BRAFV600E* mutations (~20%) who overexpress microRNAs targeting the TGF-β pathway maintains the expression of iodine metabolism genes and would be susceptible to the effects of RAI [40]. Current molecular testing techniques are still not optimized to distinguish this *BRAFV600E* subgroup in clinical practice. In contrast to the vast majority of patients with *BRAFV600E* mutations, patients with RAS mutations or with fusions exhibit a lower MAP kinase transcriptional output, which results in preservation of the expression of the sodium iodine symporter on the cell membrane and TPO that facilitates iodine uptake and retention [41].

The implications of these findings are that BRAF-like tumors will have a decreased concentration and response to RAI whereas RAS-like tumors are likely to respond to RAI and be susceptible to repeated doses of RAI therapy. For example, approximately 80% of tall-cell variant papillary thyroid carcinomas harbor a *BRAFV600E* mutation. These tumors have a high frequency of cervical lymph node metastases and loco-regional recurrences, are frequently FDG-avid on PET, and respond less to radioactive iodine due to the downregulation of iodine metabolism genes [42]. When cervical lymph node recurrences arise on a tall-cell variant papillary thyroid carcinoma which has been previously treated with adjuvant RAI, attempting to control the disease with a second dose of RAI is unlikely to be successful. Similarly, patients with *BRAFV600E* mutations and low–intermediate-risk tumors with a few lymph node metastases are unlikely to derive disease-free survival benefit from adjuvant RAI therapy. These logical hypotheses are yet to be confirmed in prospective randomized trials. In contrast, RAS mutant tumors are typically follicular thyroid carcinomas or follicular variant papillary thyroid carcinomas which spare neck lymph nodes, are prone to vascular invasion, and retain the ability to respond to radioactive iodine. Presumably, these tumors would be amenable to repeated doses of radioactive iodine. A recent study on RAS-mutated nodules with indeterminate cytology showed that only 33% of these are malignant upon immediate surgical resection and only 9.1% of the total are at an intermediate or high risk of recurrence, suggesting that by and large, the majority of RAS-mutated nodules surgically resected are benign or at low-risk of recurrence [43]. This underscores the importance of integrating pathologic characteristics with molecular markers. Only in the presence of a RAS-mutated thyroid cancer with extensive vascular invasion or other aggressive features would an adjuvant dose of RAI be justified.

In addition to primary driver mutations explaining differentiation states of thyroid tumors, we now understand that more advanced forms of the disease including high-grade papillary thyroid carcinoma and poorly differentiated thyroid carcinoma have a higher frequency of mutations in the TP53, TERT promoter, PI3K/AKT/mTOR pathway effectors, SWI/SNF subunits, and histone methyltransferases [44]. The latter two seem to lock cells in an undifferentiated state that makes them resistant to redifferentiation attempts [45]. Moreover, in a study of exceptional responders to RAI, non-responders were enriched with *BRAFV600E* mutations, chromosome 1q-gain and mutations of genes regulating mRNA splicing and the PI3K pathway. If intermediate-risk tumors are genotyped upfront, and these mutations are found, administering an adjuvant dose of RAI would only result in side effects without improved recurrence risk or survival.

Lastly, oncocytic thyroid carcinomas (OTC, previously called Hurthle cell thyroid cancer) are genetically distinct with loss of heterozygosity (LOH) in most chromosomes and uniparental disomy in chromosomes 5 and 7. Mitochondrial DNA mutations with Complex 1 loss shifts oncocytic cells to aerobic glycolysis which explains the intense avidity that these tumors demonstrate on FDG-PET scans [46,47]. While these tumors may maintain the production of thyroglobulin in large amounts, widely invasive OTC are unlikely to respond to radioactive iodine therapy based on decreased expression of the sodium iodine symporter.

Altogether, we have improved our understanding of the molecular events that make tumors more susceptible or less likely to respond to radioactive iodine. Until molecular testing is validated as a predictor of RAI responsiveness and universally adopted, we may need to rely on limited genomic information and pathologic findings to inform our decision to administer RAI therapy. Incorporating tumor genomics as a key variable in our decision-making tree will make this process considerably more informed.

## 6. Risks of RAI Therapy

The transient effects of RAI therapy include gastrointestinal distress (up to 30%), thyroiditis with mild anterior neck pain (15%), and sialadenitis (30%). Less common effects are dry eyes, nasolacrimal gland obstruction, and bone marrow suppression [48]. RAI has been associated with hypospermia and elevated FSH in some studies, which are generally thought to be transient, recovering within 1–2 years. For females, it has been established that there is no significant difference in the rate of miscarriage, stillbirths, preterm birth, and congenital anomalies between patients exposed and not exposed to RAI or in the incidence of thyroid and non-thyroid cancers in the offspring of patients who had received RAI and the general population [49,50].

When controlling for other treatment exposures, radioactive iodine confers a modest increased risk of secondary hematologic malignancies, particularly at higher dose exposures, as evidenced in multiple cohorts worldwide [51,52,53,54,55]. A recent analysis from the SEER registries suggests that RAI is associated with an increased early risk of acute myeloid leukemia (AML) and chronic myeloid leukemia (CML), but not of leukemias of lymphoid origin or of multiple myeloma [56]. A 2022 retrospective study by Pasqual et al. using SEER data from more than 40 years found that RAI was associated with increased risk of both solid and hematologic malignancies, especially when exposure occurred at younger ages and after two decades of follow up. At the currently administered activities of RAI, this absolute risk remains extremely low [57].

Discussing these side effects with intermediate-risk patients is mandatory and may deter some patients from receiving RAI entirely.

## 7. Patient Comorbidities and Preferences

Given that RAI is exquisitely incorporated into cells that express the sodium/iodine symporter, many of the typical comorbidities that preclude the use of chemotherapy agents do not affect the decision to use RAI. The use of RAI in patients with thyroid eye disease has been associated with nearly doubling of the risk in the exacerbation of Graves orbitopathy [58]. While there is some data suggesting that prophylactic steroids can reduce this risk, safety guidelines and standardized dosing have not been established [59]. Patients with thyroid cancer and concomitant malignancies like lymphoma or head and neck cancers may elect to receive a dose of RAI to facilitate follow-up of their abnormal cervical lymph nodes. Conversely, patients with hematologic malignancies may elect to avoid the added radiation expose to their bone marrow.

Among the most intricate conversations surrounding the use of radioactive iodine is that of individual patient preferences. It is crucial to consider each patient’s health history, access to care, risk tolerance, and family circumstances when making this decision. Patients must clearly understand that there is no proven benefit on recurrence or survival from this treatment in intermediate-risk patients and shared decision making should be framed within this context.

Patients are equally deterred by the logistics of radioactive iodine as they are by its adverse effects. Namely, frequent appointments over the course of a week, the decision between costly recombinant human TSH injections and a period of levothyroxine withdrawal-induced fatigue, and an isolation period during which they cannot care for small children and cannot attend work, all impact the ultimate decision to receive RAI. Further, the current recommendation is that pregnancy should be deferred for at least 6 months to 1 year after RAI therapy, and breastfeeding is absolutely contraindicated, which poses difficulties for female patients in their childbearing years.

The above emphasizes the need for shared decision making in this context. Wallner et al. showed that more than half of patients who receive radioactive iodine felt they did not have a choice in the matter. A lack of shared decision making is classically associated with more aggressive treatment/overtreatment of cancers with a favorable prognosis [60].

## 8. Integrating Key Elements to Inform Our Decision to Use RAI in Intermediate-Risk Patients

The purpose of RAI therapy should be clearly conveyed to the patient with thyroid cancer. RAI may be indicated for (a) remnant ablation, to destroy residual normal thyroid tissue and facilitate initial staging and detection of recurrent disease by thyroglobulin measurements and cross-sectional imaging studies during surveillance; (b) adjuvant therapy, intended to improve disease-free survival in patients with microscopic disease (not detected on conventional imaging studies) or without proven but suspected residual disease, and is typically given to patients with intermediate or high-risk of recurrence; or (c) therapy, to destroy thyroid cancer tissue in patients with persistent disease who are at high risk of progression [12].

We propose the integration of key variables to arrive at a treatment decision. This starts by better defining a patient’s risk of recurrence within the ATA intermediate-risk category, and by combining this information with a post-surgical thyroglobulin level. If the patient has a low–intermediate risk of recurrence and their thyroglobulin level is low, RAI may not be justified since its use may not lower the risk of recurrence, and ablating normal thyroid tissue for the purpose of thyroglobulin surveillance may not be necessary (Table 1 No RAI). If the patient has a low–intermediate-risk tumor and a high post-surgical thyroglobulin level, RAI may be used for ablative purposes. In the case of intermediate–high-risk patients, a high post-surgical thyroglobulin level may indicate persistent disease and the clinician may administer RAI for adjuvant or treatment purposes. If a post-surgical thyroglobulin level is low in an intermediate–high-risk patient, the clinician will have to decide whether the genomics of the tumor are such that the tumor is a low thyroglobulin producer and therefore unlikely to respond to RAI (No RAI), whether all disease has been successfully resected and RAI would be used for ablative purposes to follow thyroglobulin levels upon surveillance, or whether microscopic disease is suspected and therefore an adjuvant dose of RAI is indicated (Table 1).

The next essential step is critically examining the genetic mutation profile and pathologic features of the individual tumor to understand if these impact the tumor’s ability to take up and retain RAI. When available, BRAF-like tumors are less likely to respond to RAI while RAS-like tumors will have preservation of iodine metabolism genes and may be more likely to respond to a single or multiple doses of RAI.

**Figure 1:**
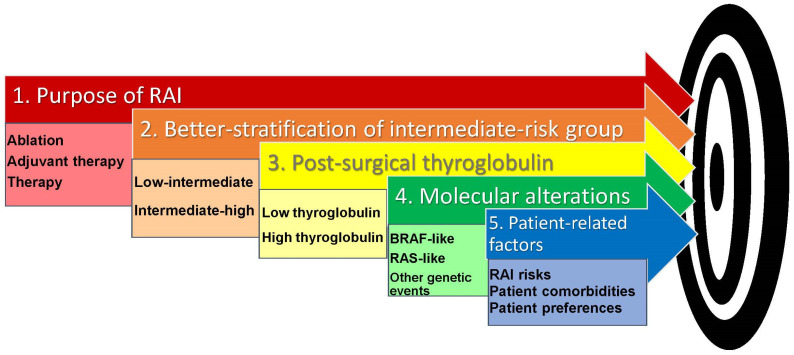
Key variables in the decision-making process for RAI use in patients with thyroid cancer at intermediate risk of recurrence.

A careful assessment of patients’ comorbidities, a detailed discussion of risks, and the patient’s preferences ultimately define the decision to proceed with RAI therapy (Figure 1).

## 9. Conclusions

In this review, we have attempted to highlight key elements that inform the decision-making process when considering RAI therapy for the management of patients with thyroid cancer at intermediate risk of recurrence. Until better molecular testing tools become clinically available and prospective randomized trials are carefully designed, the above stepwise process will result in more informed decisions that minimize overtreatment and harm.

The purpose of RAI therapy should be discussed with the patient. The decision-making process starts with a better stratification of the ATA intermediate-risk category into low–intermediate and intermediate–high based on the predicted risk of recurrence. This is combined with an evaluation of the post-surgical thyroglobulin level which predicts a low risk of persistent/recurrent disease if it is <2.5 ng/mL. Molecular alterations, when available, should be included in the decision to administer RAI as BRAF-like tumors are less likely to respond to RAI while RAS-like tumors are more susceptible to RAI therapy. Finally, RAI risks, patient comorbidities and patient preferences should all be taken into account.

## Figures and Tables

**Table 1:** Stepwise application of variables that impact use of RAI in intermediate-risk patients.

Better Stratification	Low-Intermediate Risk		Intermediate-High Risk	
**Examples**	PTC < 4 cm Minimal ETE ≤5 LN involved (0.3–3 cm)		PTC > 4 cm >5 LN involved (0.3–3 cm), Extensive vascular invasion	
**Post-surgical Tg level**	Tg < 2.5 ng/mL	Tg > 2.5 ng/mL or Tg Abs	Tg < 2.5 ng/mL	Tg > 2.5 ng/mL
**Purpose of RAI**	NO RAI	Ablation	NO RAI/Ablation/Adjuvant	Adjuvant/Therapy
**Molecular alterations**	BRAF-like/RAS-like/other molecular alterations			
**RAI risks**	Patient comorbidities			
	**Patient preferences**			
